# A Novel Magnetic Nanoparticle Drug Carrier for Enhanced Cancer Chemotherapy

**DOI:** 10.1371/journal.pone.0040388

**Published:** 2012-10-08

**Authors:** Xu Chao, Zhuoli Zhang, Lili Guo, Jingjing Zhu, Mingli Peng, Alphonsus J. M. Vermorken, Wim J. M. Van de Ven, Chao Chen, Yali Cui

**Affiliations:** 1 The College of Life Sciences, Northwest University, Xi'an, China; 2 National Engineering Research Center for Miniaturized Detection Systems, Xi'an, China; 3 Department of Radiology, Northwestern University, Chicago, Illinois, United States of America; 4 Laboratory for Molecular Oncology, Department of Human Genetics, K.U. Leuven, Leuven, Belgium; 5 The College of Basic Medical Science, Shaanxi University of Chinese Medicine, Xianyang, China; Case Western Reserve University, United States of America

## Abstract

**Background:**

Magnetic nanoparticles (NPs) loaded with antitumor drugs in combination with an external magnetic field (EMF)-guided delivery can improve the efficacy of treatment and may decrease serious side effects. The purpose of this study was 1) to investigate application of PEG modified GMNPs (PGMNPs) as a drug carrier of the chemotherapy compound doxorubicin (DOX) *in vitro*; 2) to evaluate the therapeutic efficiency of DOX-conjugated PGMNPs (DOX-PGMNPs) using an EMF-guided delivery *in vivo*.

**Methods:**

First, DOX-PGMNPs were synthesized and the cytotoxicity of DOX-PGMNPs was assessed *in vitro*. Second, upon intravenous administration of DOX-PMGPNs to H22 hepatoma cell tumor-bearing mice, the DOX biodistribution in different organs (tissues) was measured. The antitumor activity was evaluated using different treatment strategies such as DOX-PMGPNs or DOX-PMGPNs with an EMF-guided delivery (DOX-PGMNPs-M).

**Results:**

The relative tumor volumes in DOX-PGMNPs-M, DOX-PGMNPs, and DOX groups were 5.46±1.48, 9.22±1.51, and 14.8±1.64, respectively (each *p*<0.05), following treatment for 33 days. The life span of tumor-bearing mice treated with DOX-PGMNPs-M, DOX-PGMNPs, and DOX were 74.8±9.95, 66.1±13.5, and 31.3±3.31 days, respectively (each *p*<0.05).

**Conclusion:**

This simple and adaptive nanoparticle design may accommodate chemotherapy for drug delivery optimization and *in vivo* drug-target definition in system biology profiling, increasing the margin of safety in treatment of cancers in the near future.

## Introduction

Conventional chemotherapeutic compounds are distributed nonspecifically in the body, thus indiscriminately affecting both normal healthy cells as well as rapidly proliferating cancer cells. For this reason, for the desired therapeutic dose to reach the tumor, side effects occur in most cases due to high toxicity levels. The lack of targeting specificity of conventional chemotherapeutic agents makes magnetic nanoparticles (MNPs) attractive drug nanocarriers. For example, chemotherapeutic compounds may be conjugated with MNPs and can be specifically targeted to localized tumors by an external magnetic field (EMF)-guided delivery *in vivo* [1], [2]. MNPs are being extensively investigated for use as drug carriers [3], [4]. An EMF is placed and focused over the target site (i.e., the tumor or tumors). The direction of the EMF force allows nanoparticle/therapeutic agent complexes that are administered via intravenous or intra-artery injection to enter the tumor area. The EMF-guided drug enhances localized therapeutic efficacy of conjugated drugs and decreases systemic toxicity [1], [4], [5].

Gold nanoparticles have been used as carriers to investigate tumor, targeted drug delivery and laser treatment of cancer because of its better compatibility and circulation [6]–[8]. On the other hand, MNPs cooperated with polymer have been proved to be an attractive carrier as drug delivery vehicle [9]. These MNPs could not only couple drug on its surface, but also response to an EMF. Fe_3_O_4_/Au nanoparticles (GoldMag NPs/GMNPs) have a core/shell structure that is synthesized by the reduction of Au^3+^ with hydroxylamine in the presence of Fe_3_O_4_ [10], [11]. As a result, GMNPs become magnetized, thus making particles responsive to subsequently applied magnetic fields due to the Fe_3_O_4_ core. Moreover, biomolecules (e.g., antibodies, antigens, and some chemotherapeutic compounds) can be readily coupled to the surface of these GMNPs without the need for additional cross-linkers [12]–[16]. Plasma proteins adsorbed on nanoparticles are rapidly removed by the reticuloendothelial system (RES) [17]–[20]. The nanoparticles can be coated with hydrophilic polymers, such as polyethylene glycol (PEG), which are used to disperse drug particles in order to increase their half-life in the blood and to minimize or prevent protein adsorption, thus avoiding RES clearance [21], [22]. PEG is a flexible hydrophilic polymer that can be used as a shell-forming segment. The dense PEG shell allows for a high degree of biocompatibility and also endows the micelle with a stealth character in the blood compartment, consequently achieving a long circulation time [21]–[23]. PEG-modified GMNPs (PGMNPs) have been synthesized and characterized as drug carriers, which have a saturated magnetization of 34 emu/g with an average diameter of 50 nm and were homogeneous suspension without aggregation in PBS [12].

Doxorubicin (DOX)-based chemotherapy exhibited only a modest antitumor activity with tolerable adverse effects in patients with advanced hepatocellular carcinoma (HCC) [24]. The purposes of our study were 1) to investigate the kinetics of DOX-PGMNPs conjugation and the release of DOX from DOX-PGMNPs *in vitro*; 2) to assess the cytotoxicity of the DOX-PGMNPs conjugate using a mouse hepatoma cell line (H22) *in vitro*; and 3) to evaluate the therapeutic efficiency of the DOX-PGMNPs conjugate using an EMF-guided delivery and H22 hepatoma cell tumor-bearing mice *in vivo*.

## Materials and Methods

### Mouse hepatoma H22 cell line and tumor-bearing mouse model

All animals were housed and handled according to the Northwest University Institutional Animal Care and Use Committee guidelines and all animal work was approved by the appropriate committee (IACUC 0000125 and 0000125B-4). The protocol was approved by the local ethics committee (ethics committee, Northwest University 035/2009) and all animals received humane care in compliance with “The Principles of Laboratory Animal Care” formulated by the National Society for Medical Research and the “Guide for the Care and Use of Laboratory Animals” published by the National Institutes of Health (NIH Publication No. 86-23, revised 1996).

The mouse hepatoma H22 cell line was grown in RPMI-1640 medium supplemented with 10% FBS, 100 units/ml of penicillin and 100 µg/ml of streptomycin at 37°C and 5% CO_2_. BALB/c mice (4- to 8-week-old males, body weight 26±5 g) were housed (one mouse per cage) under specific pathogen-free conditions, maintained on a 12-hour light-dark cycle with controlled temperature (20–24°C), and were given sterile food. The tumor-bearing mouse model was generated by subcutaneous injection of 4×10^5^ H22 cells with 50 µl phosphate-buffered saline (PBS) into the shaved right flank. The treatment protocol required the mice to be anesthetized and these were anesthetized with ketamine (80 mg/kg) and xylazine (3 mg/kg), and acepromazine (2 mg/kg) via intraperitoneal injection (IP). If an additional dose was needed, ketamine alone was used.

### Synthesis of DOX-PGMNPs conjugates

PGMNPs were synthesized and characterized as drug nanocarriers as previously described. PGMNPs with the diameter averaging ∼50 nm were used in this study [12]. Approximately 0.2 ml of PGMNPs (10 mg/ml) suspensions was added to 5 ml centrifuge tubes, and magnetically separated with a magnetic separator (GoldMag Nanobiotec, Xi'an, China); the supernatant was discarded. A total of 2 ml of varying DOX concentrations (0.1, 0.2, 0.3, 0.4, 0.5, 0.6 mg/ml) was added to the PGMNPs precipitate. The mixtures were incubated in a shaker at room temperature for 4 hrs. The DOX-PGMNPs conjugate was magnetically separated and the drug content left in the supernatant was measured with high-performance liquid chromatography (HPLC, Shimadzu 2010A instrument, Shimadzu, Japan). HPLC analysis was performed using a binary pump, column oven, and UV detector. LC solution software was used for the data analysis. The chromatographic separation was performed on an Inertsil ® ODS-SP analytical column (150 mm×4.6 mm, 5 µm, Shimadzu, Japan) with the column temperature set at 25°C. An isocratic elution was performed after 15 min using 0.1% acetic acid and acetonitrile in the ratio 72/28 (v/v). The flow rate was 0.8 ml/min, the detector wavelength was set to 254 nm, and the injection volume was 20 µl. The drug loading (%) on the surface of the PGMNPs was calculated using the following equation: Drug loading (%) = (the mass of drug on PGMNPs/the mass of PGMNPs)×100%.

### DOX release study *in vitro*

A total of 5 mg of the DOX-PGMNPs conjugate (DOX loading 8.2%) was suspended in 15 ml PBS (pH 7.4). The releasing medium was placed in a shaking incubator at 37±0.5°C and 180 rpm for 15 min. PGMNPs were then separated, and an aliquot of the supernatant (0.5 ml) was retained. The concentration of DOX in the supernatant was quantified by HPLC and the amount of DOX released from the PGMNPs was calculated. Drug release (%) from the DOX-PGMNPs conjugate was calculated at different time points using the following equation: Drug release (%) = (mass of drug in the supernatant/mass of drug originally conjugated onto the PGMNPs)×100%.

### Cytotoxicity of PGMNPs and the DOX-PGMNPs conjugate in H22 cell culture

A volume of 180 µl of H22 cells were seeded in 96-well plates (4,000 cells/well). The following day, 20 µl DOX (0.4, 4, 8, 20 or 40 µg/ml) or 20 µl DOX-PGMNPs conjugate (4.88, 48.8, 97.6, 244 or 488 µg/ml) was added to H22 cell suspensions (DOX loading rate 8.2%). In both cases, the final concentration of DOX was the same (0.04, 0.4, 0.8, 2 and 4 µg/ml). The cells were treated with various DOX concentrations (from DOX solution or DOX-PGMNPs conjugate) in 96-well plates at 37°C and 5% CO_2_ atmosphere for 24 hrs.

The cytotoxicity of equivalent amounts of PGMNPs (compared to DOX-PGMNPs conjugate) was evaluated as well. Control cells were cultured in medium only. Thiazolyl blue tetrazolium bromide (25 µl of 5 mg/ml) (MTT, AMRESCO Inc, Solon, OH) was added to each well and incubated for 4 hrs. The culture medium was removed from the wells and replaced with 150 µl dimethylsulfoxide (DMSO, BioTek, Winooski, VT). After shaking gently for 15 min at 25°C, the DMSO solution was transferred to centrifugation tubes and centrifuged at 2,000 rpm for 5 min. The absorbance was then measured at 570 nm with an ELx-800 Universal Microplate Reader (BioTek, Winooski, VT). The cell inhibition rate was calculated using the formula: cell inhibition rate = (1−A_Test_/A_Control_)×100%; where A_Test_ is the absorbance of the experimental wells and A_Control_ is the absorbance of the control wells. The half-maximal inhibitory concentration (IC_50_; drug concentration corresponding to 50% growth inhibition) was calculated using SigmaPlot 9.0 software (Systat Software Inc., San Jose, CA) and the 4-parameter logistic function standard curve analysis for dose response.

### DOX biodistribution in tumor-bearing mice and chemotherapy experiments

The H22 tumor-bearing mice were randomly divided into 4 groups; Group I (DOX group) was injected with 0.15 ml DOX dissolved in physiologic saline solution (0.82 mg/ml); Group II (DOX-PGMNPs group) was injected with 0.15 ml DOX-PGMNPs conjugate suspension (10 mg PGMNPs/ml, DOX loading 8.2%); Group III (DOX-PGMNPs-M group) was injected with 0.15 ml DOX-PGMNPs conjugate suspension (10 mg/ml, DOX loading 8.2%) using a 0.5 tesla EMF (0.5 T permanent magnet, Northwestern Polytechnical University, Xi'an, China) applied on the tumor for 2 hrs; and Group IV (Control group) was injected with 0.15 ml physiologic saline solution alone to serve as a control. The doses were normalized to 5 mg DOX per kg body weight when the tumor volume reached 50 to 100 mm^3^ (∼10 days after tumor cell inoculation). The volume for 150 µl was injected via the tail vein and the treatment was carried out three times without interval in three weeks.

DOX biodistribution measurement: mice (Group I, Group II, and Group III; n = 6 for each group) were sacrificed after 0.5 hr of treatment for measurement of DOX biodistribution. The blood, main organs (heart, lung, liver, spleen, kidney), and the tumor of each mouse were harvested for quantitative analysis of the DOX biodistribution. The blood was centrifuged at 5,000 rpm for 1 min at 4°C and the supernatant was obtained by adding 180 µl methanol and 20 µl of inner standard solution (0.2 mg/ml daunorubicin solution dissolved in methanol) to a 2-ml tube containing 200 µl plasma. For the organ (heart, lung, liver, spleen, kidney) and tumor samples, the frozen tissues were weighed and homogenized at 4°C in 0.9% NaCl to a total volume of 10 ml, and centrifuged at 5,000 rpm for 1 min at 4°C to retrieve the supernatant.

A total volume of 200 µl of the plasma, or the supernatant of homogenized tissue, or the tumor samples was then mixed with 180 µl methanols and 20 µl inner standard solutions, followed by 0.4 g sodium sulfate. The mixture was homogenized by vortexing for 5 min and then incubated at 4°C for 1 hr. After the 1-hr incubation, the mixture was centrifuged at 12,000 rpm for 15 min. The supernatant (50 µl) was analyzed by HPLC and the quantity of DOX in the tissue and tumor samples was measured. Finally, the percent-injected dose per gram tissue of the tissue and tumor samples was calculated.

Chemotherapy experiments: H22 tumor-bearing mice (4 groups; for each group, n = 18) were used and the treatment was performed as described above. The antitumor effect was assessed by the following steps: 1) six mice were sacrificed for each group on the 24^th^ day following treatment. The tumors were harvested and fixed in 10% formalin, embedded in paraffin and sectioned (5 µm coupes) for hematoxylin–eosin (HE) staining for microscopic examination purposes; 2) tumor sizes (4 groups; for each group, n = 12) were measured by a caliper before therapy and every three days after treatment using the following equation: tumor volume (V, mm^3^) = (tumor length×tumor width)^2^/2. The life span of each animal was also recorded. The relative tumor volume was calculated from V = V/V_0_, where V_0_ is the tumor volume at the beginning of treatment. The endpoints of the antitumor effect were evaluated by the degree of tumor growth inhibition and life span after treatment.

### Statistical analysis

Quantitative data were expressed as the mean ± standard deviation (SD). The means were compared using Student's t test where *p* values<0.05 were considered statistically significant.

## Results

### Efficiency of DOX loading on the surface of PGMNPs

**Figure 1:**
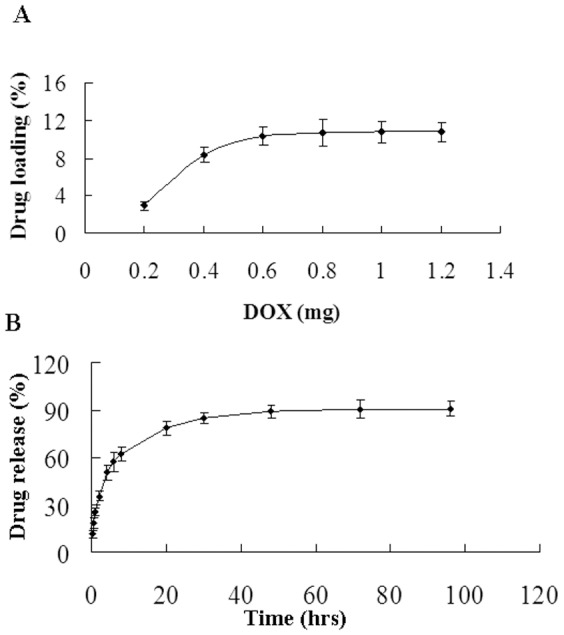
Efficiency of DOX loading onto the surface of PGMNPs and the behavior of DOX release from DOX-PGMNPs conjugates. (**A**) Efficiency of DOX loading onto the surface of PGMNPs was analyzed by HPLC. The amount of DOX conjugated to PGMNPs was positively correlated with the DOX mass added. (**B**) DOX release from DOX-PGMNPs conjugates was analyzed by HPLC. The slow, steady, and controlled release of DOX was observed.

The amount of DOX conjugated to 2 mg PGMNPs was directly proportional to the amount of DOX added. The DOX loading ranged from 2.98% to 10.78% depending on the amount of DOX used (0.2 to 1.2 mg). The loading rate achieved saturation when DOX exceeded 0.6 mg (Fig. 1A).

### DOX release from DOX-PGMNPs conjugates *in vitro*

The cumulative DOX release from DOX-PGMNPs conjugate is shown in Figure 1B. In the first 0.5 h, 20.4% of DOX was released while 60.1% of DOX was released in 8 hrs. Subsequently, the DOX release amounts were 79.3%, 90.2%, and 91.3% at 20 hrs, 48 hrs, and 72 hrs, respectively. The maximum DOX release (92.2%) from the surface of the PGMNPs was achieved at 100 hrs.

### Cytotoxicity of PGMNPs and DOX-PGMNPs

**Figure 2:**
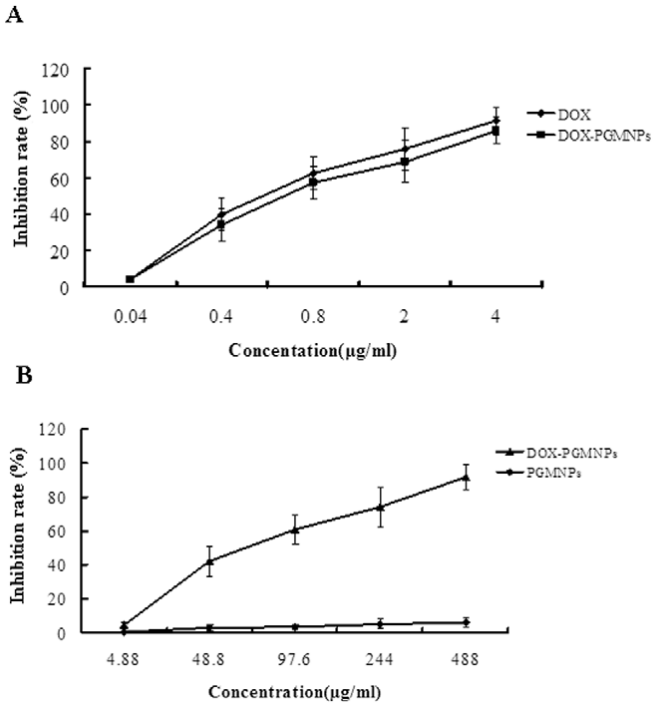
Cytotoxicity profiles of PGMNPs, DOX and DOX-PGMNPs upon H22 cell lines upon H22 cell lines determined by MTT assay. (**A**) H22 cells suspension were treated by DOX or DOX-PGMNPs conjugate. (**B**) H22 cells suspension were treated by PGMNPs or DOX-PGMNPs conjugate.

The cell inhibition rate was determined using the MTT assay and the results are shown in Figure 2. The cytotoxicity of DOX and DOX-PGMNPs presented in Figure 2A, which shows that the cell inhibition rate increased with increasing drug concentration. The cell inhibition rate of DOX was modestly higher than that of the DOX-PGMNPs group (IC_50_ values were 0.530±0.010 µg/ml and 0.652±0.056 µg/ml (*p*>0.05), respectively). Moreover, when compared with DOX-PGMNPs group, PGMNPs alone did not affect H22 hepatoma cell proliferation/viability (Fig. 2B).

### DOX biodistribution in tissues

**Figure 3:**
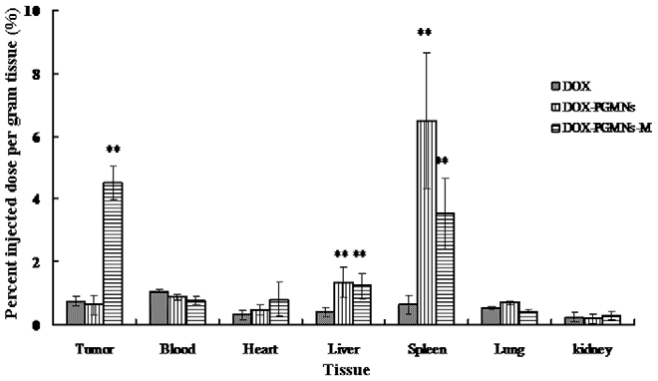
The percent injected dose per gram tissue in tumor, plasma and main organs of H22 tumor–bearing mice that received different treatments. The percent injected dose per gram tissue in the tumors of mice in the DOX-PGMNPs-M group was much higher than that in tumors of mice in the DOX-PGMNPs group or the DOX group. DOX(H22 tumor-bearing mice that treated with doxorubicin; DOX-PGMNPs (H22 tumor-bearing mice that treated with DOX-PGMNPs conjugate suspension); DOX-PGMNPs-M (H22 tumor-bearing mice injection DOX-PGMNPs conjugate suspension and subjected to a 0.5 tesla EMF focused onto the tumor for 0.5 hr).*p<0.05; **p<0.01 compared with controls using Student's unpaired t-test.

The percent injected dose per gram tissue in tumors of mice from the DOX-PGMNPs-M group, DOX-PGMNPs group, and DOX group were 4.51%, 0.627% and 0.741%, respectively (Fig. 3). Therefore, our results show that DOX concentrations in tumors of mice from the DOX-PGMNPs-M group were significantly higher than those from the DOX-PGMNPs group or the DOX group (each *p*<0.05). The percent injected dose per gram tissue in blood of mice from the DOX-PGMNPs-M group, the DOX-PGMNPs group, and the DOX group were 0.765%, 0.876%, and 1.07%, respectively (for each group *p*>0.05). The percent injected dose per gram tissue in livers from the DOX-PGMNPs-M group, the DOX-PGMNPs group, and the DOX group were 1.23%, 1.33% and 0.402%, respectively. Therefore, no significant difference was observed between the DOX levels found in mouse livers of the DOX-PGMNPs and DOX-PGMNPs-M groups (*p*>0.05). However, the liver DOX concentration appeared to be much lower in mice of the DOX group compared with mice in the DOX-PGMNPs-M or DOX-PGMNPs groups (each *p*<0.05). Interestingly, the highest DOX concentrations were found in the spleen. The percent injected dose per gram tissue in from mouse spleens were 3.52%, 6.49% and 0.634% reported from the DOX-PGMNPs-M, the DOX-PGMNPs, and the DOX groups, respectively (each *p*<0.05). Very low concentrations of DOX were observed in the other organs (e.g., the heart, lung and kidney) for all three groups (DOX, DOX-PGMNPs, and DOX-PGMNPs-M).

### Histological studies

**Figure 4:**
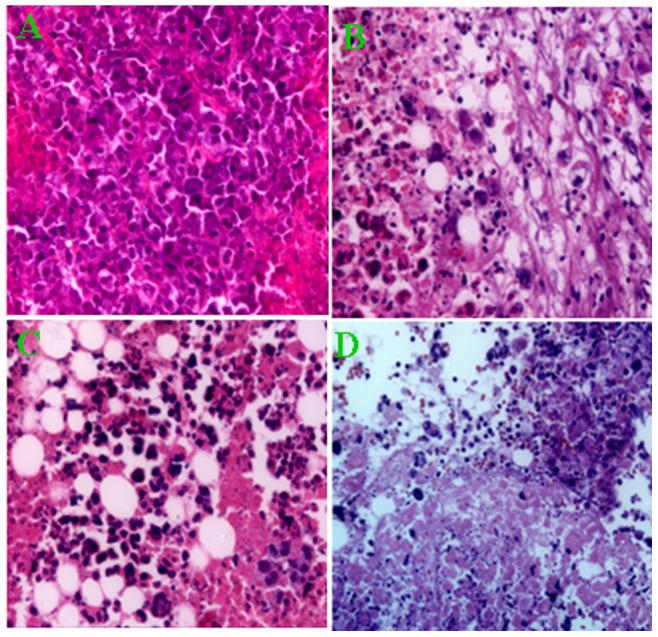
Histological assay of tumors of H22 tumor-bearing mice that received different treatments. (hematoxylin and eosin stain, 400× magnifications). A large number of necrotic cells as well as vacuoles were observed in tumors in mice of the DOX-PGMNPs-M group (D), whereas only a few necrotic tumor cells could be seen in tumors in mice of the DOX (B) or DOX-PGMNPs groups (C). Mostly viable tumor cells were observed in the tumors of the mouse control group (A).

Tumor tissue was investigated using HE staining. Most necrotic cells and vacuoles were observed in tumors from the DOX-PGMNPs-M group. A few necrotic cells were observed in tumors from the DOX and DOX-PGMNPs groups, while only viable tumor cells were observed in tumors from the control group (Fig. 4).

### Influence of DOX-PGMNPs on tumor growth

**Figure 5:**
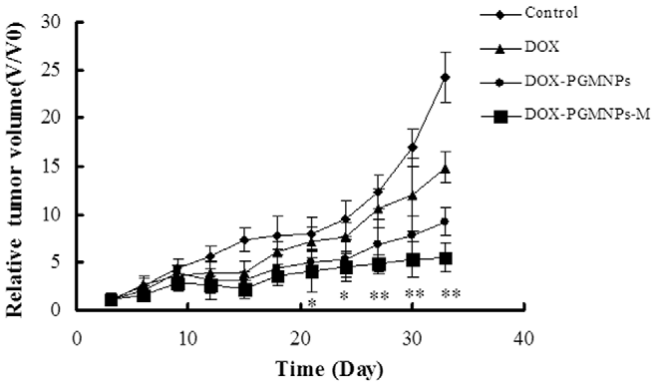
Tumor growth curves of H22 tumor –bearing mice model that received different treatments indicated. Effective inhibition of the tumor growth can be seen resulting from the DOX-PGMNPs and DOX-PGMNPs-M treatment strategies. The same doxorubicin dose was injected to H22 tumor –bearing mice model for DOX, DOX-PGMNPs and DOX-PGMNPs-M. The same volume physiologic saline solution injected to H22 tumor –bearing mice model as control. *p<0.05; **p<0.01 compared with controls using Student's unpaired t-test.

Tumor growth curves resulting from different drug treatments of H22 hepatoma cell tumor-bearing mice are shown in Figure 5. The relative tumor volume of DOX-PGMNPs-M group, DOX-PGMNPs group, DOX group and the control group were determined 21 days after the start of various treatment protocols; they were 3.98±2.12, 4.96±1.45, 7.08±1.68 and 7.92±1.73, respectively. At 21 day, the DOX-PGMNPs-M treatment provided an effective inhibition of tumor growth that was comparable to those found for the other treatment strategies (*p*<0.05). It should be emphasized that at this time point (at 21 days), there was no significant difference between the effective inhibition of tumor growth in the DOX group and the DOX-PGMNPs group (*p*>0.05). However, at day 33 (after the start of the various treatment protocols), the inhibition of tumor growth was greatest in the DOX-PGMNPs-M group. The relative tumor volume in the DOX-PGMNPs-M group was 5.46±1.48, in the DOX-PGMNPs group was 9.22±1.51, in the DOX group was 14.8±1.67, and in the control group was 24.3±1.95 (for each *p*<0.01). It should be noted that a significant difference in the fractional percent of tumor volume was observed between the groups treated with DOX, with DOX-PGMNPs and with DOX-PGMNPs-M (*p*<0.05).

### Influence of DOX-PGMNPs on life span

**Figure 6:**
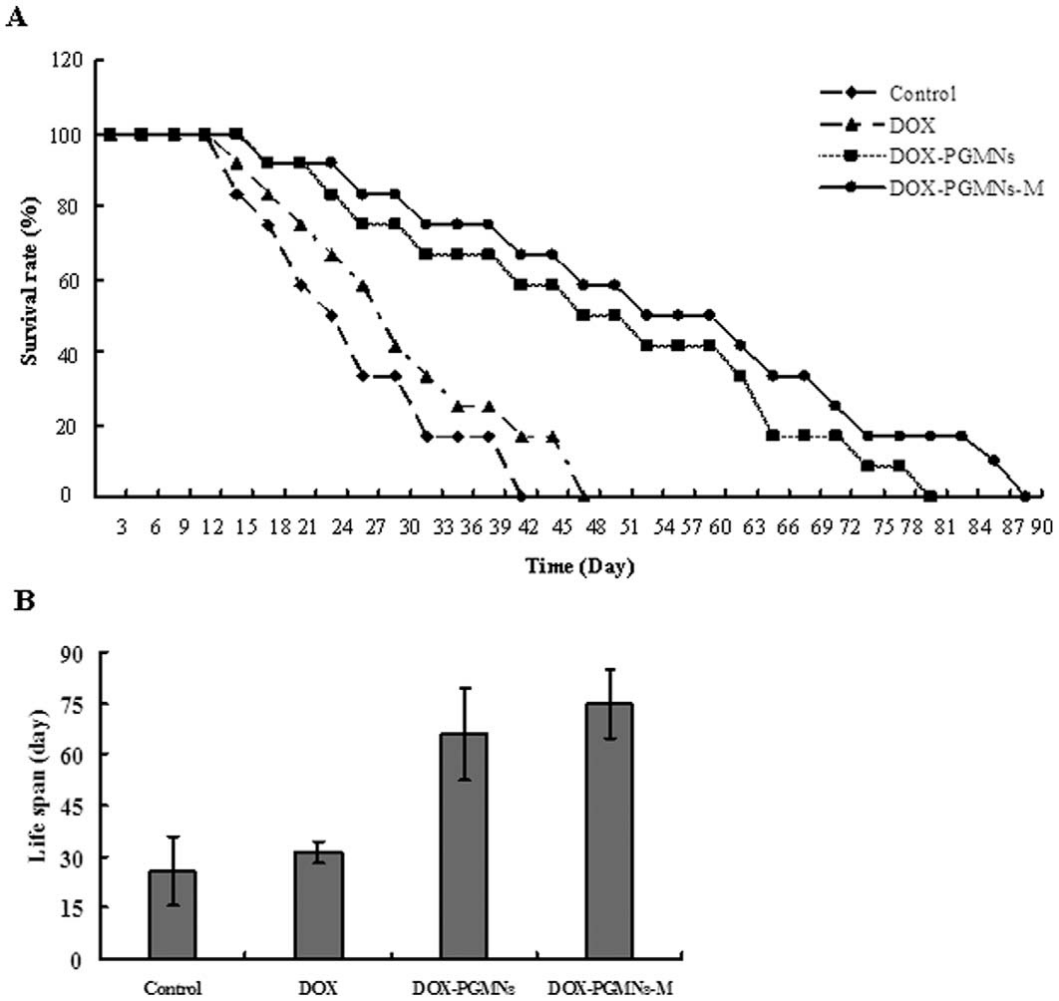
The survival as a function of time and life span of H22 tumor-bearing mice model that received different treatments. (A) The survival as a function of time of the tumor-bearing mice of the DOX-PGMNPs-M group. (B)There is a significantly increased life span of the tumor-bearing mice of the DOX-PGMNPs-M group.

The survival rate of tumor-bearing mice treated with DOX-PGMNPs-M, DOX-PGMNPs, DOX and the control group decreased with the time. The survival rate of tumor-bearing mice treated with DOX-PGMNPs-M, DOX-PGMNPs, DOX and the control group were 75%, 66.7%, 33.3% and 16.7% following treatment for 33 days (Fig. 6A).

The life span of tumor-bearing mice treated with DOX-PGMNPs-M, DOX-PGMNPs, DOX and the control group were 74.8±9.95, 66.1±13.5, 31.3±3.31 and 25.8±10.1 days, respectively (each *p*<0.05) (Fig. 6B). The life span of mice in the DOX-PGMNPs group and the DOX-PGMNPs-M group was significantly longer than of those in the DOX group or in the control group (each *p*<0.05). Furthermore, the life span of mice in the DOX-PGMNPs-M group was significantly longer than of those in the DOX-PGMNPs group (*p*<0.05).

## Discussion

It is very critical for the drug adsorbed on the particles can release from carrier. Also the ideal drug delivery carrier is one from which the drug can release with a sustained and controlled amount within given time. The loading and release patterns of DOX from PGMNPs showed that DOX could be efficiently loaded onto and released from PGMNPs (Fig. 1). The DOX loading ranged from 2.98% to 10.78% with the amount of DOX increase and the amount of maximum drug loading rate is about 107.8 µg/mg, which drug loading is 10.78% (Fig. 1A). The loading rate achieved saturation when the concentration exceeded 0.6 mg/ml. The results of drug release studies *in vitro* showed a period of rapid release in the first 10 hrs and drug release saturation after 20 hrs (Fig. 1B). Because of –OH group in DOX and PEG, the drug release behaviors are affected by the pH value and temperature. Based on the data, it was proved that the key interaction of DOX and PEG molecules modified on gold-magnetic nanoparticles is the hydrogen-bond [12]. In addition, the drug release behavior is in accord with the toxicity results of free DOX and DOX-PGMNPs in H22 hepatoma cells after 24 hrs exposure. Although the cell inhibition rate of DOX is modestly higher than that of DOX-PGMNPs, our studies show that DOX- PGMNPs display a similar toxicity profile as free DOX in H22 cells (Fig. 2A), thus indicating that DOX-PGMNPs have sufficient antitumor activity to inhibit tumor growth.

*In vitro* cytotoxicity assays showed that PGMNPs were not significantly cytotoxic because 85.4% of H22 cells cultured in the presence of 2.0 mg/ml remained viable (Fig. 2B). The lack of any significant cytotoxicity being observed may be explained by the following: 1) the NPs have a gold shell and colloid gold is known to have low toxicity and good biocompatibility [25], [26]; and 2) PEG is a biocompatible hydrophilic polymer that can improve the properties of NPs by decreasing their toxicity [27], [28]. The cytotoxicity of DOX-PGMNPs was studied *in vitro* and the IC_50_ values of free DOX and DOX-PGMNPs were found to not be significantly different. We assume the cause that cell inhibition rate in DOX is lightly higher than the DOX-PGMNPs group is the DOX-PMGNPs exist first the release of DOX from the NPs; although it could not reach the inhibition effect as the same drug level as the free DOX group. Some DOX-PMGNPs could be uptaken by cells that led to potent cytotoxicty than free DOX as the same quality as which loaded in PMGNPs. Both factor are attributed to the cytotoxcity of DOX-PMGNPs are lower than DOX, although they had no significantly difference statistically. These results demonstrated that DOX-PGMNPs are potent cytotoxins towards H22 hepatoma cancer cells (Fig. 2A).

Our results (from various treatments) indicated that the concentration of DOX was significantly lower in mouse livers compared with spleens (Fig. 3). Previous reports have shown that PEG-coated gold nanoparticles are accumulated in both the spleen and the liver [29], [30]. Although the amount of nanoparticles should correlate with the amount of DOX in the system, this finding could be explained by the fact that DOX may be metabolized in the liver and, therefore, may be causing lower amounts of the compound to be detected in the liver *in vivo*.

Magnetically targeted drug delivery system could improve therapy efficacy by increasing the drug concentration in the tumor while reducing systemic drug concentrations that produce systemic toxicities [31], [32]. Antitumor activity of the DOX-PMGPNs-M treatment was observed by determining the relative tumor volume and the mouse life span (Figs. 5 and 6). The therapeutic potency and efficacy of the DOX-PGMNPs-M treatment was demonstrated by tumor necrosis. The antitumor activities might be attributed to the various drug concentrations in the tumors due to the differences in the experiments (Figs. 3, 5 and 6). The DOX-PGMNPs-M therapy showed a significant increase of DOX efficiency when compared with DOX therapy alone, which is most likely because PGMNPs effectively carry DOX to the targeted site (tumor) by an EMF force. DOX concentrations in tumors of mice from the DOX-PGMNPs-M group were significantly higher than those from the DOX-PGMNPs group or the DOX group after injection 0.5 hr. This led to the therapeutic potency and efficacy of the DOX-PGMNPs-M. In contrast, there was no difference of DOX concentration in the tumors between the groups treated with DOX or DOX-PGMNPs (Fig. 3). This could attributed to enhanced permeability and retention (EPR) effect of DOX-PGMNPs in the liver and spleen and the drug controlled release behavior. Therefore, it did not lead to a higher DOX accumulation in the tumor of DOX-PGMNPs compared with free DOX. The data of the concentration of DOX in the livers, tumors and spleen of DOX-PGMNPs also is according to the results. The efficiency of tumor growth inhibition was substantially more pronounced in mice of the DOX-PGMNPs group than in mice of the DOX group, although there have no significantly difference between DOX-PGMNPs-M group and DOX-PGMNPs-m group. This could imply that the growth-inhibitory effect of the DOX-PGMNPs (Figs. 5 and 6) is not only related to the DOX concentration but also to the DOX-releasing behavior from DOX-PGMNPs *in vivo*. Although our studies on H22 cells indicated that PGMNPs displayed no significant toxicity (or minor toxicity) *in vitro*, a direct cytotoxic effect of the PGMNPs on the tumor cells *in vivo* cannot be excluded. The data of life span of mice treated with DOX-PGMNPs-M, DOX-PGMNPs, and DOX (74.8±9.95, 66.1±13.5, and 31.3±3.31 days) were showed that there is significantly efficiency for tumor treatment of DOX-PGMNPs group compared to the DOX group. Using an EMF to locate these drug-loading carriers, the efficiency for tumor treatment will be better.

In summary, DOX-PGMNPs binding and releasing were efficient *in vitro* investigations and the results show that most of DOX-PGMNPS were cleared by RES and targeted to liver and spleen. This indicates that the PGMNPs constitute highly attractive nanocarriers and offer a promising perspective for an EMF-guided delivery of anticancer therapeutics and integration in cancer treating protocols. Although additional research is needed to further demonstrate that PGMNPs are indeed general drug carriers, the possibility that a single carrier could be used to transport multiple classes of drugs to their desired targets makes these PGMNPs versatile therapeutic tools. Furthermore, pharmacokinetic profiles obtained to date certainly warrant further investigation of these PGMNPs. This technology has the potential to provide the long-felt need for a precise, consistent, and efficient drug delivery method that can be used in numerous medical applications.
